# Sex‐ and Region‐Specific Glial Reactivity in Hyperthyroid Mice Lacks Correlation With the Noncognitive and Non‐Depressive‐Like Behavioral Alterations

**DOI:** 10.1002/brb3.71501

**Published:** 2026-05-29

**Authors:** Qianqian Xue, Zefang Lin, Yaofeng Wang, Xiuting Lin, Yun Yang, Chao Wang, Yuhong Ye, Weibing Miao

**Affiliations:** ^1^ Department of Nuclear Medicine, National Regional Medical Center, Binhai Campus of the First Affiliated Hospital Fujian Medical University Fuzhou Fujian China; ^2^ Department of Nuclear Medicine, The First Affiliated Hospital Fujian Medical University Fuzhou Fujian China; ^3^ Department of Pathology, The First Affiliated Hospital Fujian Medical University Fuzhou Fujian China; ^4^ Department of Nuclear Medicine, Provincial Clinical Key Specialty of Fujian, The First Affiliated Hospital Fujian Medical University Fuzhou Fujian China; ^5^ Fujian Key Laboratory of Precision Medicine for Cancer, The First Affiliated Hospital Fujian Medical University Fuzhou Fujian China

**Keywords:** astrocytes, cognitive, depression, glial reactivity, hyperthyroidism, microglia, PET/CT

## Abstract

**Background:**

Glial reactivity is implicated in hyperthyroidism‐associated cognitive and psychiatric disorders, yet in vivo imaging evidence of glial reactivity in hyperthyroidism remains to be elucidated. This study aimed to detect hyperthyroidism‐induced glial reactivity using ^1^ ^8^F‐DPA714 positron emission tomography/computed tomography (PET/CT) imaging and investigate the associations with behavioral alterations in mice.

**Methods:**

C57BL/6J mice were randomly divided into hyperthyroid (T4) and control groups. ^1^ ^8^F‐DPA714 PET/CT imaging quantified glial reactivity as standardized uptake value (SUV) in eight brain regions. Immunohistochemistry for ionized calcium‐binding adapter molecule 1 (IBA‐1) and glial fibrillary acidic protein (GFAP) validated glial reactivity in CA1 pyramidal layer of the hippocampus and layer IV of the somatosensory cortex. Behavioral tests included sucrose preference, forced swim, and water maze.

**Results:**

Cortical and hippocampal IBA‐1 and GFAP densities were significantly elevated in T4 mice, with sex‐dependent GFAP expression (male higher in cortex, female higher in hippocampus). PET/CT showed that T4 markedly increased SUV in striatum, thalamus, hypothalamus, brainstem, and midbrain in female mice only, while in cortex, hippocampus, and amygdala, T4 increased SUV in both sexes. Hyperthyroid mice did not show cognitive decline or depressive‐like behaviors. Instead, male T4 mice displayed shortened immobility time, and both sexes showed increased platform crossings and greater target quadrant distance. No significant associations were found between glial reactivity measures and behavioral outcomes.

**Conclusions:**

Hyperthyroidism induces sex‐ and region‐specific glial reactivity detected by ^1^ ^8^F‐DPA714 PET/CT and pathology, which lacks correlation with the observed noncognitive and non‐depressive‐like behavioral alterations in mice.

## Graphical abstract

Hyperthyroidism induces sex‐ and region‐specific glial reactivity without correlation to the noncognitive, non‐depressive behavioral changes in mice.

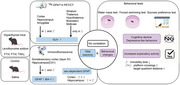

## 1 Introduction

Essential roles of thyroid hormones (THs) in sustaining the normal development of the central nervous system (CNS) have been well documented (Anderson et al. 2003; Bernal 2005; Remaud et al. 2014). Thyroid disorders in adults are associated with psychiatric and cognitive problems (Joffe and Sokolov 1994; Wu et al. 2016; Winkler et al. 2016; Correia et al. 2009). Hyperthyroidism can induce many clinical symptoms, including emotional lability, impatience and irritability, distractible overactivity (Awad 2000), depression, and anxiety (Demet et al. 2002). Extensive research has revealed that hyperthyroidism is correlated with an elevated risk of dementia (George et al. 2019; Kalmijn et al. 2000; Döbert et al. 2003; Mooradian and Haas 2025; Benseñor et al. 2010).

There are many possible biological mechanisms through which hyperthyroidism is involved in cognitive and psychiatric problems (Tang et al. 2021; Shahat et al. 2022; Bianchi et al. 1999; Villanueva et al. 2013; Jurado‐Flores et al. 2022). Inflammatory stress is considered one of the mechanisms (Bianchi et al. 1999; Villanueva et al. 2013; Jurado‐Flores et al. 2022). Upon exposure to inflammatory stress, glial cells—especially astrocytes and microglia—and certain nonneural cellular populations exhibit morphological remodeling and altered protein expression profiles, while secreting a variety of inflammatory cytokines (Honda et al. 2001). In addition, TH can lead to reactive microglia and astrocytes and affect microglial functions via complex mechanisms (Mori 2014; Mori et al. 2015; Noda 2015; Woodburn et al. 2021). Neuroglial effects may help to elucidate physiological and pathophysiological functions of TH in CNS.

Identifying an imaging method that can objectively assess glial reactivity in vivo is essential for further exploring the clinical value of glial reactivity. ^18^F‐DPA714 positron emission tomography/computed tomography (PET/CT) imaging targeted the 18‐kDa translocator protein (TSPO) has been used to evaluate and monitor glial reactivity in many diseases (Papadopoulos et al. 2006; Cosenza‐Nashat et al. 2009; Loggia 2024; Boniek and Malamut 2025; Mueller et al. 2023). However, whether glial reactivity actually occurs in the hyperthyroid brain and whether it contributes to the associated behavioral deficits remain unknown.

In this study, we therefore tested the hypothesis that hyperthyroidism induces glial reactivity in the brain, which correlates with cognitive and affective alterations. To this end, we used ^1^ ^8^F‐DPA714 PET/CT imaging combined with pathological analysis to detect and quantify glial reactivity in hyperthyroid mice. The correlation between glial reactivity and behavioral alterations was analyzed to investigate the role of glial reactivity, with partialled by sex in groups of male and female mice.

## 2 Materials and Methods

### 2.1 Hyperthyroid Mouse Model

The experimental cohort consisted of C57BL/6J mice aged 6 weeks, which were obtained from Sibeifu Biotechnology. All experimental animals were acclimatized to the housing environment for 1 week before the formal experiment initiation. Following this acclimation period, the mice were allocated into either a hyperthyroid group or a euthyroid control group via a randomized design. Mice received levothyroxine sodium to induce hyperthyroidism. The levothyroxine (L‐T4) sodium‐induced hyperthyroidism group (T4 group, *n* = 30) and the euthyroid control group (*n* = 30) had an equal sex distribution.

The housing facility maintained a strictly regulated environment where the ambient temperature was stabilized at 26°C and the relative humidity was kept between 40% and 70%. Lighting followed a standardized 12‐h diurnal cycle, with illumination occurring between 8:00 a.m. and 8:00 p.m. To minimize social isolation stress, the animals were housed in groups of five per cage and granted unrestricted access to a standard laboratory diet and potable water throughout the investigative timeframe.

Levothyroxine sodium was obtained from Merck Pharmaceuticals (Jiangsu) Co. Ltd. The reagent was prepared by dissolving the compound in a sterile physiological saline solution to achieve a final concentration of 35 µg/mL. For a period of 11 consecutive days, mice in the T4 group received daily subcutaneous injections at a standardized volume of 0.1 mL per 10 g of body mass (Leal et al. 2007). Control subjects were administered an equivalent volume of the saline vehicle via the same subcutaneous route to ensure that any observed physiological shifts were specifically attributable to the hormone itself rather than the physical stress of the injection procedure.

Twenty‐four hours after the last levothyroxine sodium injection, eight mice (four control mice, four hyperthyroid mice) used for serum level detection were spared from behavioral testing. After confirming the successful modeling, behavioral tests were performed on each group of 20 mice (10 males, 10 females). After behavioral tests, PET/CT imaging was performed on five male and five female mice randomly selected from each group. The remaining sex‐matched mice were deeply anesthetized by intraperitoneal injection of tiletamine‐zolazepam (Zoletil 50, 110–150 mg/kg) combined with xylazine hydrochloride (Sumianxin, 30–50 mg/kg). Euthanasia was carried out via cardiac puncture after complete disappearance of corneal reflex and nociceptive response. Subsequently, brain tissues were rapidly dissected for subsequent pathological analysis. During behavioral tests and PET/CT imaging, levothyroxine sodium and saline were administered via subcutaneous injection to the hyperthyroid and control mice, respectively.

### 2.2 PET/CT Imaging

^1^ ^8^F‐DPA714 PET/CT imaging was carried out on a nanoScan PET/CT scanner (Mediso Ltd., Budapest, Hungary). Mice were injected with 8.0 MBq ^1^ ^8^F‐DPA714 via tail vein intravenously, followed by a 50‐min in‐cage incubation for radiotracer uptake. Prior to image acquisition, mice were anesthetized with isoflurane (4% for induction and 2% for maintenance, both in oxygen), followed by a 10‐min static PET scan and a subsequent CT scan for anatomical co‐registration. Mice were returned to cages for recovery post‐imaging, and all images were reconstructed with the 3D‐OSEM algorithm with attenuation, random, and scatter corrections.

To ensure anatomical accuracy and standardization across the experimental cohort, the reconstructed PET images were manually co‐registered to the Ma‐Benveniste‐Mirrione C57BL/6J mouse brain MRI template (PMOD v3.7, PMOD Technologies, Zurich, Switzerland), a minimum‐deformation atlas of C57BL/6J mice. Regions of interest (ROIs) were automatically extracted from the pre‐segmented Ma‐Benveniste‐Mirrione mouse brain atlas embedded in PMOD, with precise anatomical definitions as follows: (1) cortex (neocortex, all cortical regions of the cerebral hemispheres); (2) hippocampus (whole hippocampal formation, including CA1, CA3, and dentate gyrus); (3) amygdala (amygdaloid nuclear complex); (4) striatum (dorsal striatum/caudate‐putamen); (5) thalamus (whole thalamus, including all thalamic nuclei); (6) hypothalamus (medial basal hypothalamus); (7) midbrain (whole mesencephalon, including tectum and tegmentum); (8) brainstem (pons and medulla oblongata, excluding midbrain and cerebellum). Mean standardized uptake values (SUV_mean_) were calculated for the entire 3D volume of each ROI and recorded and used for statistical analysis.

The ROIs defined for the thalamus, midbrain, and brainstem in the present study were delineated as large, comprehensive anatomical regions without further subdivision into individual subnuclei or subregions. Therefore, the observed tracer uptake changes in these broad ROIs lack fine anatomical regional specificity, and potential variations within specific subnuclei may not be captured. This should be considered when interpreting the findings from these regions.

### 2.3 Measurement of TH

Mice were euthanized following the aforementioned protocol. Blood samples were collected and incubated at room temperature for 2 h. The collected blood samples were centrifuged at a speed of 1000 rpm for 20 min to isolate the serum, which was subsequently preserved at −80°C until required for analysis. Systemic concentrations of free triiodothyronine (FT3), free thyroxine (FT4), thyroid‐stimulating hormone (TSH), and thyroglobulin antibody (TGAb) were determined using enzyme‐linked immunosorbent assay kits from Elabscience in strict accordance with the protocols provided by the manufacturer.

### 2.4 Sucrose Preference Test

Sucrose preference test was performed mainly according to the previously reported method (Primo et al. 2023). Mice were kept in cages with two bottles containing 1% (w/v) sucrose solution for 1 day (Day 1). The next day (Day 2), one bottle was replaced with one containing tap water, and the left/right locations of the bottles were switched every 2 h. Then, the mice underwent 24 h of food and water deprivation. The mice were then housed individually in separate cages to freely choose to drink either 1% (w/v) sucrose solution or tap water, which were randomly placed on the left or right sides of cages for the 24‐h test (Day 4). The sugar water preference index (SWPI) was calculated by dividing sucrose solution intake by total liquid intake (sucrose solution + tap water intake).

### 2.5 Forced Swim Test

According to the previously reported method (Can et al. 2012), mice were pretrained to swim for 10 min in a cylindrical glass tank (20 cm in height, 14 cm in diameter) containing 10 cm of water maintained at 25 ± 1°C. On the following day, the mice were reintroduced to the same tank for a 6‐min test session, and the duration of immobility was recorded to assess depression‐like behavioral phenotypes.

### 2.6 Water Maze Test

Assessment of spatial learning and memory capabilities within the experimental subjects was conducted through the Morris water maze assay, including five consecutive training days (four trials per day) and a probe trial on the sixth day (Vorhees and Williams 2006). For each individual trial, a mouse was gently placed into the water from the midpoint of one of the four pool walls (facing the wall) and given a 60‐s maximum time to locate a submerged hidden platform. Any subject failing to reach the submerged platform within the designated timeframe was manually directed to its location and remained there for 5 s. For the probe trial phase, the escape platform was extracted from the pool to allow each mouse to navigate the water freely for a total of 60 s. All swimming behaviors were automatically captured and recorded by a computerized video‐tracking system (KEMaze, Nanjing Calvin Biotechnology Co. Ltd.). Key behavioral parameters, including the swimming distance in the target quadrant, average swimming speed, and the number of platform crossings, were extracted and documented for subsequent analysis.

### 2.7 Immunofluorescence

After euthanasia, whole brains were removed and post‑fixed in 4% paraformaldehyde (PFA) diluted in phosphate‐buffered saline (PBS) at 4°C for 24 h. Following fixation, the brain tissues were dehydrated in graded ethanol (70%, 80%, 90%, 95%, and 100%), cleared in xylene, and then infiltrated with molten paraffin through multiple changes to completely replace the clearing agent. The brains were then placed in embedding molds, correctly oriented in molten paraffin, and cooled to form solid paraffin blocks. The resulting brain tissue blocks were sectioned coronally at 4 µm thickness using a rotary microtome. According to the *Mouse Brain Atlas*, fifth edition (Paxinos and Franklin 2019), coronal sections containing the following target regions were selected: The CA1 pyramidal layer of the dorsal hippocampus (Bregma −1.8 to −2.3 mm) and layer IV of the primary somatosensory cortex (S1, barrel cortex) (Bregma −0.56 to −0.90 mm). Section positions were confirmed based on anatomical landmarks. The selected sections were mounted onto poly‐L‐lysine‐coated slides and baked at 60°C for 2 h.

Subsequent immunofluorescence staining was performed mainly according to a previously reported method (Li et al. 2023). Following deparaffinization and rehydration, mouse brain sections were subjected to heat‑mediated antigen retrieval in citrate buffer (pH 6.0) at 98°C for 40 min. After three washes in PBS (5 min each), sections were permeabilized with 0.5 % Triton X‑100 for 10 min and blocked with 10% normal goat serum for 1 h at room temperature. Sections were then incubated overnight at 4°C with either rabbit anti‑IBA‑1 (Proteintech, 10904‑1‑AP, 1:200) or rabbit anti‑GFAP (Proteintech, 16825‑1‑AP, 1:200). Both antibodies have been fully validated by the manufacturer via knockdown/knockout (KD/KO) assays, confirmed for specific reactivity in human, mouse, and rat brain tissues with positive tissue controls, and verified for immunofluorescence applications. In addition, these two antibodies have been widely adopted in hundreds of studies (Xia et al. 2021; He et al. 2024; Inyang et al. 2025), further supporting their high specificity and reliability. The next day, sections were incubated with Cy3‑conjugated goat anti‑rabbit IgG (H + L) secondary antibody (AS007, ABclonal, 1:200) for 1 h at room temperature. After further PBS rinses, nuclei were counterstained with DAPI for 3 min. Fluorescent signals of the hippocampal CA1 pyramidal layer and the primary somatosensory cortex were finally observed and imaged using a confocal laser scanning microscope (Carl Zeiss AG, Germany).

### 2.8 Statistical Analyses

Experimental results are represented by the mean ± standard error (SE). Statistical evaluations were performed utilizing the SPSS 27.0 software package, whereas GraphPad Prism 9 and Figdraw facilitated data visualization and the creation of scientific figures. Two‐way analysis of variance (ANOVA) was utilized to assess the effects of T4 intervention (T4 group vs. control group) and sex (male vs. female) as fixed factors on serum TH levels, body weight, food intake, behavioral test parameters, average GFAP/IBA‐1 densities, and brain region‐specific SUV values, with special attention paid to their interaction effects. In cases where a significant interaction was identified (*p* < 0.05), simple main effect analyses with Bonferroni correction were performed; if no significant interaction was observed (*p* ≥ 0.05), only the main effects of the two factors were interpreted. A *p*‐value of less than 0.05 was defined as statistically significant. For behavioral parameters showing significant differences between the T4 group and control group, Pearson correlation analysis was carried out to explore the correlations between glial reactivity and these behavioral indices. To address the issue of multiple comparisons, the Benjamini and Hochberg false discovery rate (FDR) correction was applied. Under this criterion, statistical significance was limited to correlations yielding an FDR‐adjusted *p*‐value (P‐FDR) < 0.05.

## 3 Results

### 3.1 Successful Establishment of the Hyperthyroidism Model in Mice

**FIGURE 1:**
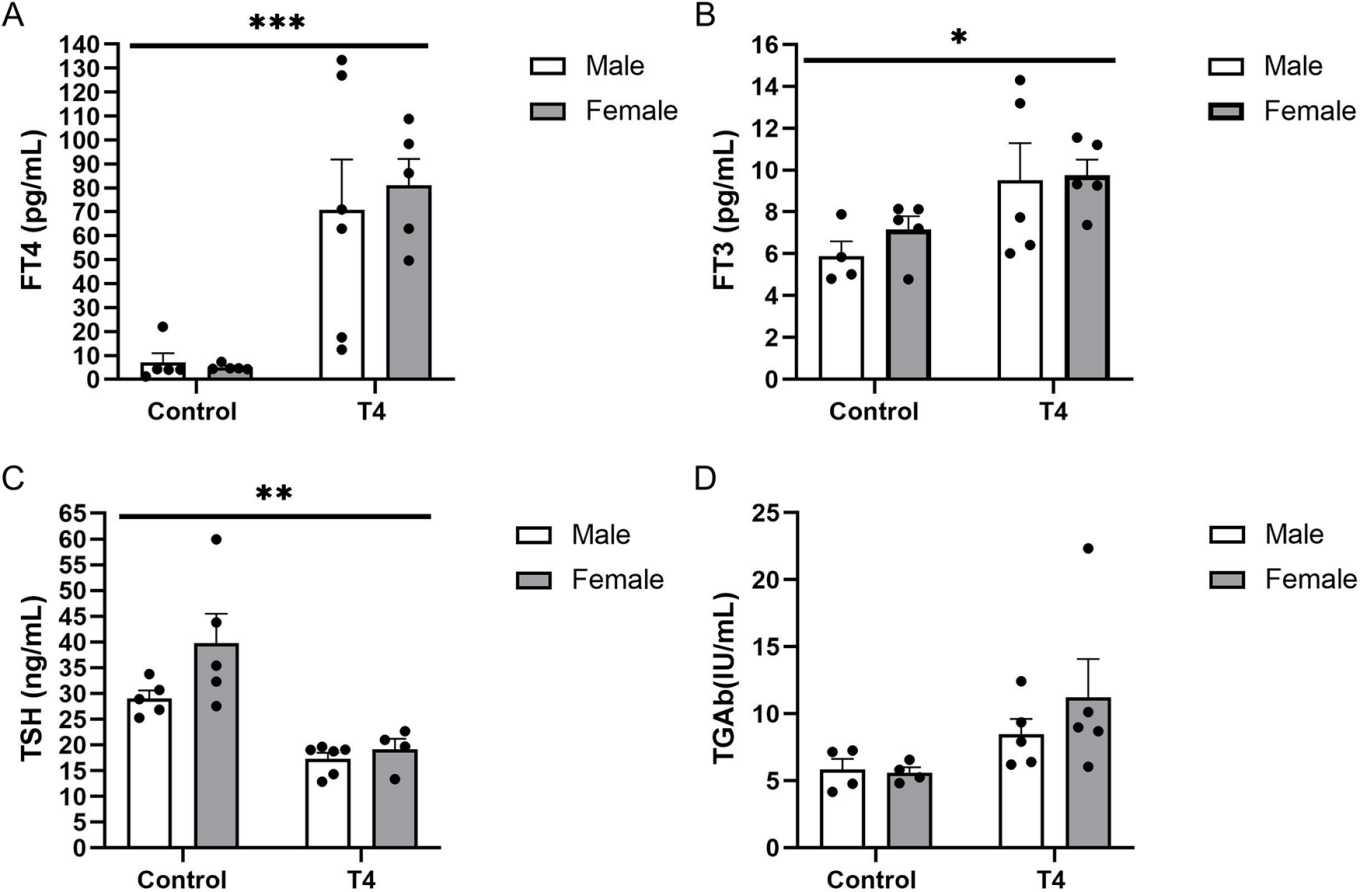
Successful establishment of the hyperthyroidism model. The levels of FT4 (*p* < 0.001), (A) and FT3 (*p* = 0.021), (B) were significantly elevated in T4 mice, while TSH levels were markedly decreased (*p* < 0.002), (C). (D) No significant difference in TGAb was observed between the two groups. *p < 0.05, **p < 0.01, ***p < 0.001.

There was no significant T4 × sex interaction detected for FT3, FT4, TSH, and TGAb levels in mice, indicating consistent effects of T4 across sexes. T4 exerted significant main effects on the three indices: FT4 (*F* = 26.921, *p* < 0.001, partial *η* ^2^ = 0.613), FT3 (*F* = 6.768, *p* = 0.021, partial *η* ^2^ = 0.326), TSH (*F* = 15.292, *p* = 0.002, partial *η* ^2^ = 0.540), and no significant main effect on TGAb. In comparison with the control mice, the T4 group had markedly elevated estimated marginal mean concentrations of FT4 (75.941 ± 9.311 vs. 6.083 ± 9.725 pg/mL; Bonferroni‐adjusted *p* < 0.001, Figure 1A) and FT3 (9.044 ± 0.683 vs. 6.531 ± 0.683 pg/mL; Bonferroni‐adjusted *p* = 0.021, Figure 1B), a significantly lower estimated marginal mean TSH level (18.230 ± 2.436 vs. 33.826 ± 3.158 ng/mL; Bonferroni‐adjusted *p* = 0.002, Figure 1C), and a marginally higher estimated marginal mean TGAb level (9.362 ± 1.289 vs. 5.452 ± 1.403 IU/mL; Bonferroni‐adjusted *p* = 0.061, Figure 1D). Sex had no significant main effect for any index.

### 3.2 No Cognitive Decline and Depression‐Like Behaviors in Hyperthyroid Mice

#### 3.2.1 Forced Swimming Test

**FIGURE 2:**
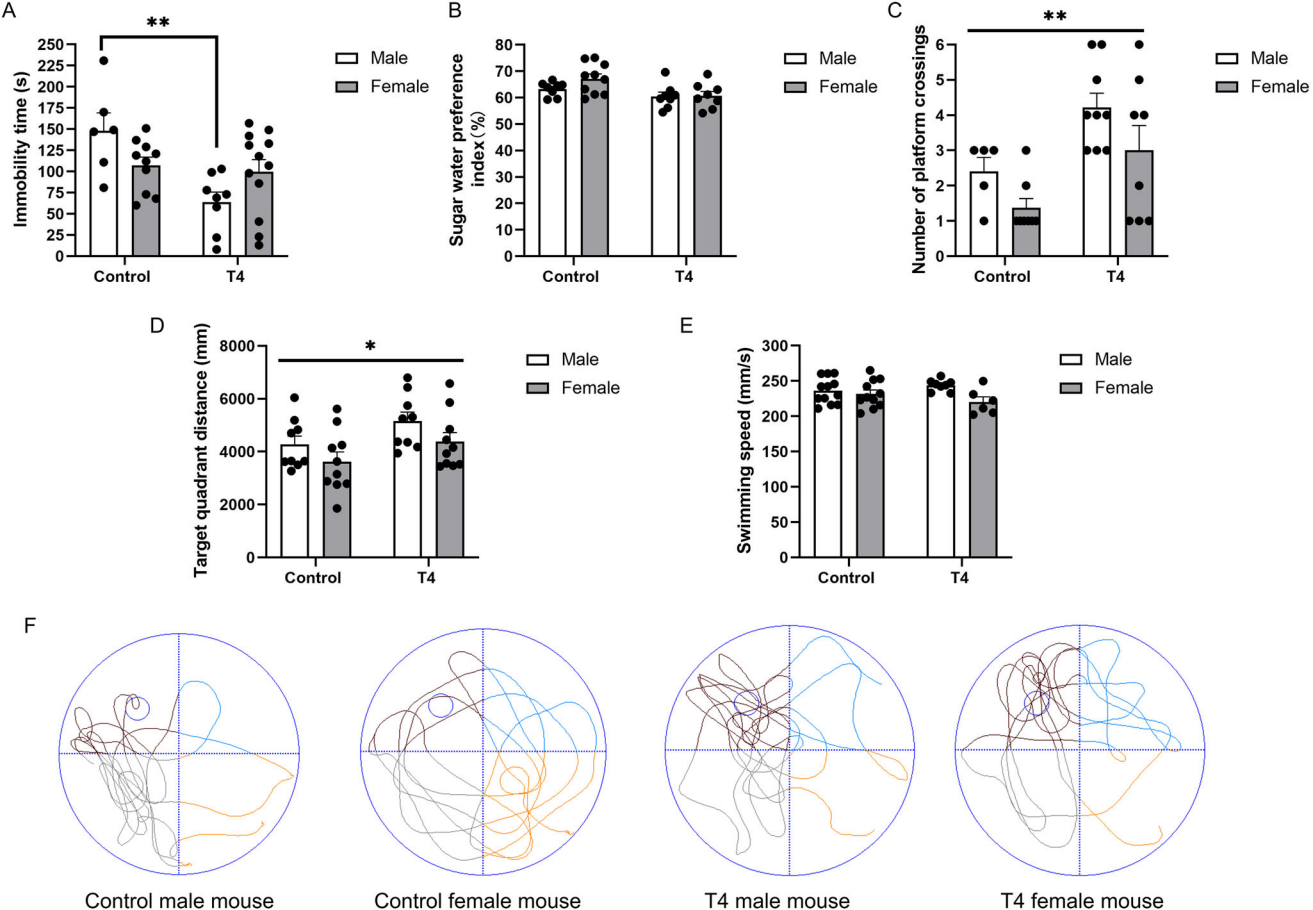
No cognitive decline and depression‐like behaviors in hyperthyroid mice. (A) Immobility time (s) was significantly shorter in male T4 mice (*p* = 0.003), with no significant difference observed in females. (B) There was no significant difference in the sugar water preference index between the control group and the T4 group. (C) The number of platform crossings was significantly increased in the T4 group (*p* = 0.002). Male mice exhibited more platform crossings than female mice (*p* = 0.034). (D) Target quadrant distance (mm) was significantly longer in the T4 group (*p* = 0.023). Mice traveled a longer target quadrant distance in males than in females (*p* = 0.048). (E) There was no significant difference in swimming speed between the control group and the T4 group. (F) Representative water maze test trajectories of the control and T4 group (male/female). Trajectories from different quadrants were plotted in different colors, with the small blue circle denoting the hidden platform. *p < 0.05, **p < 0.01.

Significant T4 × sex interaction for immobility time in the forced swim test was detected (*F* = 5.626, *p* = 0.024, partial *η* ^2^ = 0.158, Figure 2A). Simple main effect analysis showed that in male mice, immobility time was significantly shorter in the T4 group (63.750 ± 15.387 s) than in the control group (148.250 ± 21.761 s; Bonferroni‐adjusted *p* = 0.003). In contrast, in female mice, immobility time was shorter in the T4 group (99.833 ± 12.564 s) than in the control group (107.200 ± 13.763 s), without significance. Within the control group, there was no significant sex difference in immobility time. In the T4 group, male mice exhibited a marginally shorter immobility time than female mice, but this difference did not reach statistical significance.

#### 3.2.2 Sucrose Preference Test

No significant T4 × sex interaction, main effect of T4, or main effect of sex on SWPI was observed in mice (Figure 2B).

#### 3.2.3 Water Maze Test

No significant T4 × sex interaction on the number of platform crossings was detected. A highly significant main effect of T4 was detected (*F* = 11.808, *p* = 0.002, partial *η* ^2^ = 0.312). The estimated marginal mean number of platform crossings was significantly higher in the T4 group (3.611 ± 0.325) than in the control group (1.888 ± 0.382, Figure 2C). A significant main effect of sex was also observed (*F* = 5.018, *p* = 0.034, partial *η* ^2^ = 0.162). Male mice showed significantly higher estimated marginal mean number of platform crossings (3.311 ± 0.373) than female mice (2.188 ± 0.335, Figure 2C).

About the target quadrant distance, no T4 × sex interaction effect was detected, indicating that the effect of T4 on target quadrant distance was similar in male and female mice. A significant main effect of T4 was detected (*F* = 5.686, *p* = 0.023, partial *η* ^2^ = 0.143). Target quadrant distance was significantly longer in the T4 group (4772.459 ± 244.459 mm) than in the control group (3948.061 ± 244.459 mm, Figure 2D). The main effect of sex was significant (*F* = 4.190, *p* = 0.048, partial *η* ^2^ = 0.110). Male mice exhibited significantly longer target quadrant distance (4714.086 ± 250.810 mm) than female mice (4006.434 ± 237.939 mm, Figure 2D).

About the swimming speed, neither the T4 × sex interaction effect nor the main effect of T4 was significant. Swimming speed was comparable between the control group (234.595 ± 3.494 mm/s) and the T4 group (232.066 ± 4.762 mm/s, Figure 2E). In contrast, the main effect of sex was significant (*F* = 5.201, *p* = 0.029, partial *η* ^2^ = 0.136). Male mice exhibited significantly faster swimming speed (240.066 ± 4.071 mm/s) than female mice (226.595 ± 4.280 mm/s).

### 3.3 Immunofluorescence Manifestation of Cerebral Glial Reactivity in Hyperthyroid Mice

**FIGURE 3:**
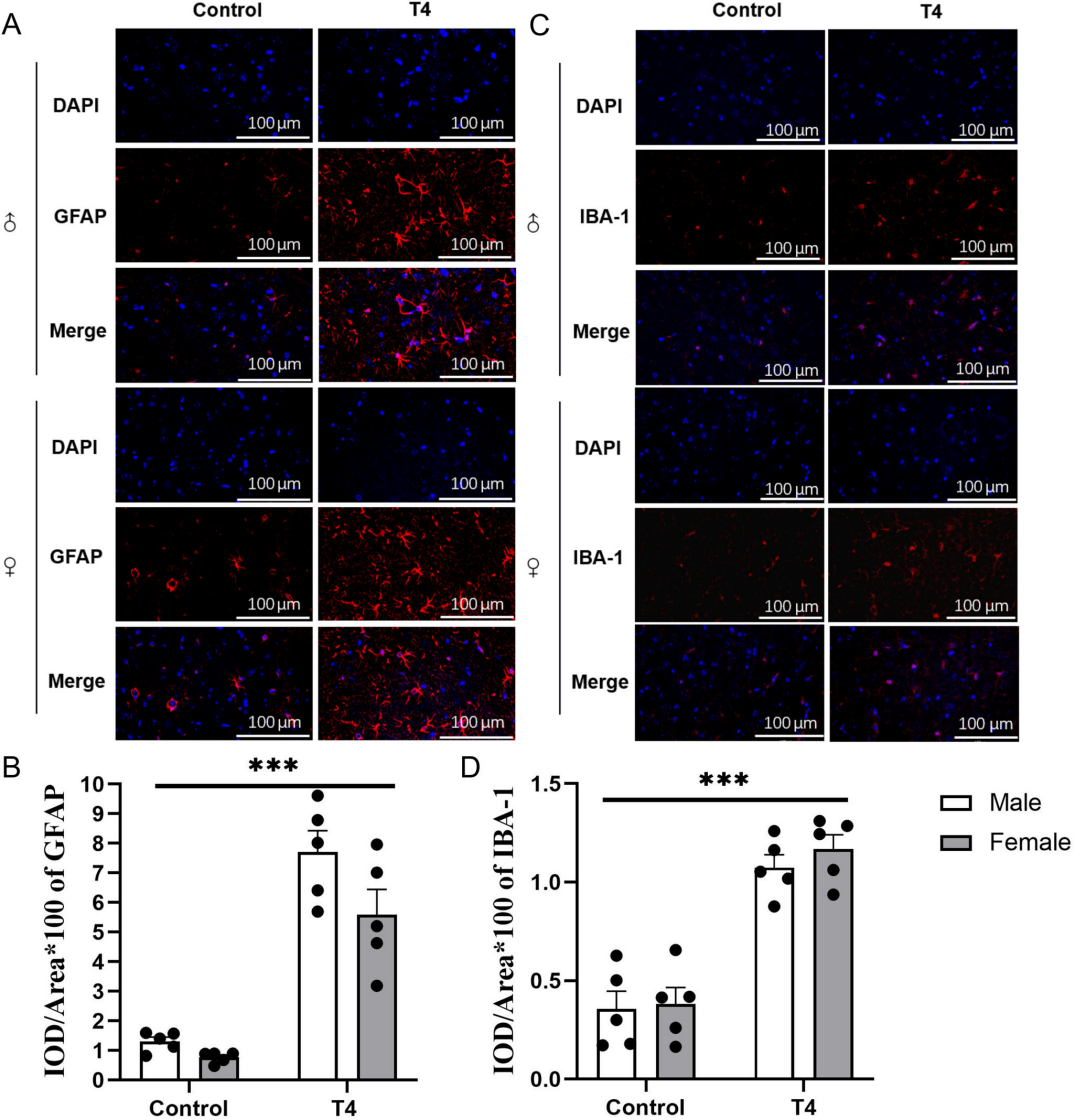
Immunofluorescence (IF) and quantification of reactive microglia and astrocytes in the cerebral cortex of hyperthyroid mice. (A) IF of GFAP expression in the cortex of mice from different groups and sexes (× 400). Scale bar, 100 µm. *N* = 20. (B) The average densities of cortical GFAP (*p* < 0.001) were significantly increased in the T4 mice. (C) IF of IBA‐1 expression in the cortex of mice from different groups and sexes (× 400). Scale bar, 100 µm. *N* = 20. (D) The average densities of cortical IBA‐1 (*p* < 0.001) were significantly increased in the T4 mice. ***p < 0.001.

**FIGURE 4:**
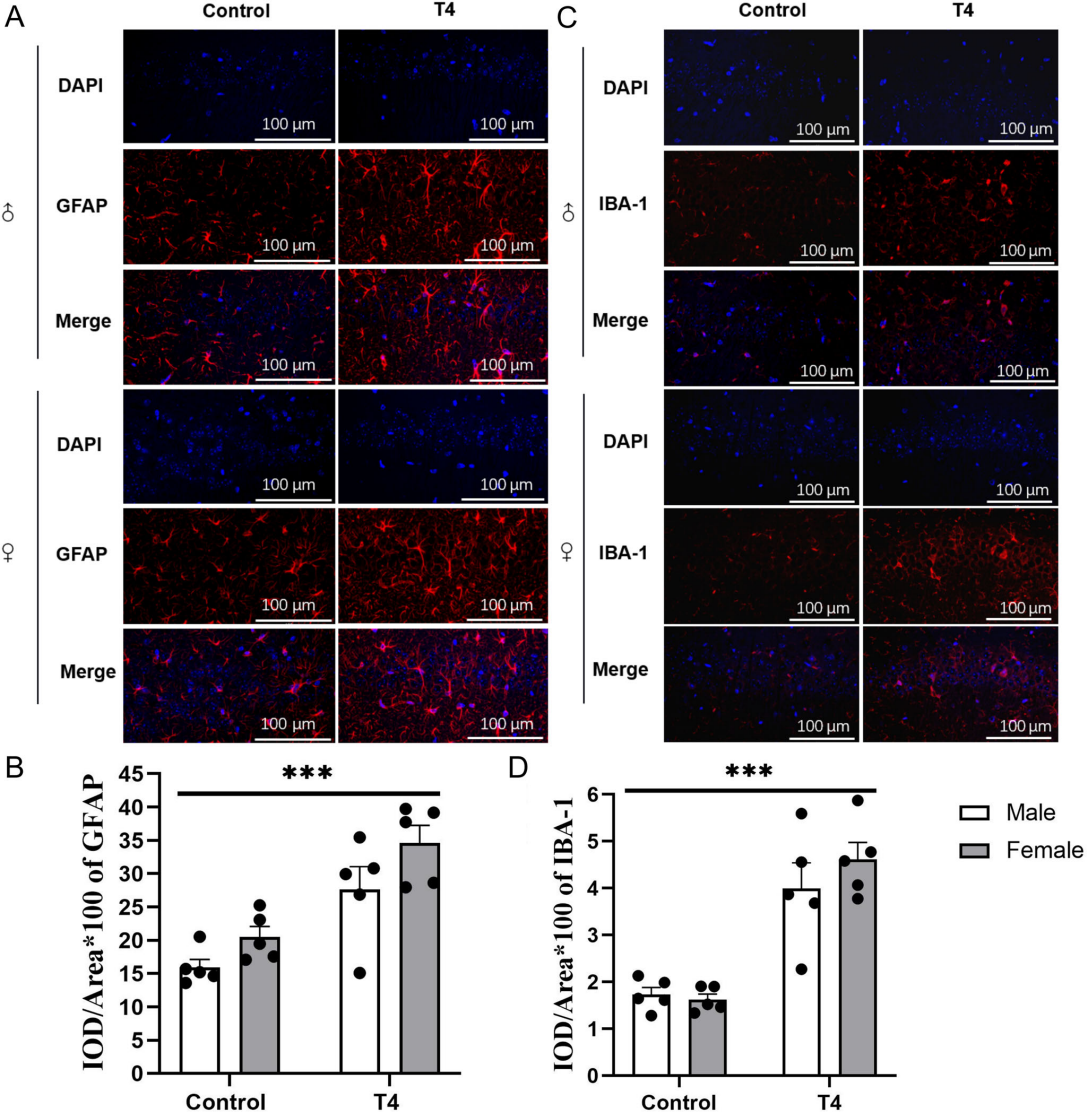
Immunofluorescence (IF) and quantification of reactive microglia and astrocytes in the hippocampus of hyperthyroid mice. (A) IF of GFAP expression in the hippocampus of mice from different groups and sexes (× 400). Scale bar, 100 µm. *N* = 20. (B) The average densities of hippocampal GFAP (*p* < 0.001) were significantly increased in the T4 mice. (C) IF of IBA‐1 expression in the hippocampus of mice from different groups and sexes (× 400). Scale bar, 100 µm. *N* = 20. (D) The average densities of hippocampal IBA‐1 (*p* < 0.001) were significantly increased in the T4 mice. ***p < 0.001.

Integrated optical density (IOD) values were acquired, and the average density (expressed as %, calculated as IOD divided by the area of the target distribution region) was computed. T4 exerted significant main effects on the four indices: cortical GFAP (*F* = 119.032, *p* < 0.001, partial *η* ^2^ = 0.68, Figure 3A,B), cortical IBA‐1 (*F* = 46.555, *p* < 0.001, partial *η* ^2^ = 0.454, Figure 3C,D), hippocampus GFAP (*F* = 66.557, *p* < 0.001, partial *η* ^2^ = 0.543, Figure 4A,B), hippocampus IBA‐1 (*F* = 124.342, *p* < 0.001, partial *η* ^2^ = 0.689, Figure 4C,D), with the T4 group exhibiting significantly higher GFAP and IBA‐1 densities than the control group. For sex effects, cortical GFAP density was higher in males (*F* = 6.579, *p* = 0.013, partial *η* ^2^ = 0.105, Figure 3A,B), hippocampal GFAP density was higher in females (*F* = 13.3, *p* = 0.001, partial *η* ^2^ = 0.192, Figure 4A,B), and no significant sex differences were found for cortical or hippocampal IBA‐1 densities. No significant treatment × sex interactions were detected for any measure, confirming that T4‐induced glial reactivity in the cortex and hippocampus was consistent across sexes (See Table 1 for details).

**TABLE 1:** Two‐way ANOVA analysis of GFAP and IBA‐1 average density in the mice cerebral cortex and hippocampus.

Average density of GFAP/IBA‐1	Effect type	*F*	*p*	Partial *η* ^2^	Intergroup comparison results (Bonferroni‐adjusted)
GFAP_ cortex	Main effect of T4	119.032	< 0.001	0.68	T4 group: 6.651 ± 0.364 control group: 1.036 ± 0.364
GFAP_ cortex	Main effect of sex	6.579	0.013	0.105	Males: 4.504 ± 0.364 Females: 3.184 ± 0.364
GFAP_ cortex	T4 × sex interaction effect	2.328	0.133	0.04	—
IBA‐1_cortex	Main effect of T4	46.555	< 0.001	0.454	T4 group: 1.121 ± 0.078 Control group: 0.369 ± 0.078
IBA‐1_ cortex	Main effect of sex	0.295	0.589	0.005	—
IBA‐1_ cortex	T4 × sex interaction effect	0.093	0.761	0.002	—
GFAP_ hippocampus	Main effect of T4	66.557	< 0.001	0.543	T4 group: 31.145 ± 1.121 Control group: 18.217 ± 1.121
GFAP_ hippocampus	Main effect of sex	13.3	0.001	0.192	Females: 27.570 ± 1.121 Males: 21.791 ± 1.121
GFAP_ hippocampus	T4 × sex interaction effect	0.594	0.444	0.01	—
IBA‐1_ hippocampus	Main effect of T4	124.342	< 0.001	0.689	T4 group: 4.305 ± 0.166) Control group: 1.680 ± 0.166
IBA‐1_ hippocampus	Main effect of sex	1.186	0.281	0.021	—
IBA‐1_ hippocampus	T4 × sex interaction effect	2.378	0.129	0.041	—

*Note*: Data are presented as the mean ± S.E. All statistical analyses were performed using two‐way ANOVA. Effect sizes were quantified by partial *η* ^2^ (partial eta‐squared), with the following classifications: small effect (0.01–0.06), medium effect (0.06–0.14), and large effect (> 0.14). Statistical significance was set at *p* < 0.05.

Abbreviations: GFAP, glial fibrillary acidic protein; IBA‐1, ionized calcium‐binding adapter molecule 1.

### 3.4 In Vivo PET/CT Imaging Visualizes Cerebral Glial Reactivity in Hyperthyroid Mice

**FIGURE 5:**
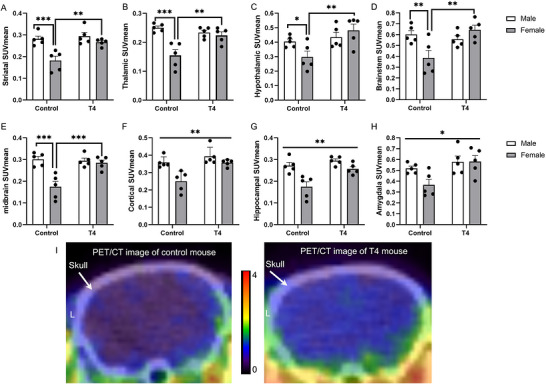
In vivo imaging of glial reactivity in hyperthyroid mice. The SUVmean in the striatum (A, *p* = 0.001), thalamus (B, *p* = 0.001), hypothalamus (C, *p* = 0.002), brainstem (D, *p* = 0.001), and midbrain (E, *p* < 0.001) were significantly increased in female T4 mice, but not in T4 males. Control males exhibited significantly higher SUV values in these regions than control females: striatum (A, *p* < 0.001), thalamus (B, *p* < 0.001), hypothalamus (C, *p* = 0.049), brainstem (D, *p* = 0.005), and midbrain (E, *p* < 0.001), whereas no significant sex differences were observed in T4 mice. The SUV_mean_ in the cortex (*p* < 0.002, F), hippocampus (*p* < 0.004, G), and amygdala (*p* < 0.010, H) were significantly increased in the T4 mice. (I) Representative axial ^18^F‐DPA714 PET/CT images of control and T4 mice. Color scale: SUV_mean_ 0.0 (*darkest*)–4.0 (*brightest*); warmer colors = higher radiotracer uptake. *p < 0.05, **p < 0.01, ***p < 0.001.

Significant T4 × sex interactions were detected in the SUV_mean_ of striatum (*F* = 5.406, *p* = 0.034, partial *η* ^2^ = 0.253, Figure 5A), thalamus (*F* = 11.584, *p* = 0.004, partial *η* ^2^ = 0.420, Figure 5B), hypothalamus (*F* = 4.703, *p* = 0.046, partial *η* ^2^ = 0.227, Figure 5C), brainstem (*F* = 10.421, *p* = 0.005, partial *η* ^2^ = 0.394, Figure 5D), and midbrain (*F* = 12.186, *p* = 0.003, partial *η* ^2^ = 0.432, Figure 5E). T4 significantly increased SUV_mean_ in female mice across these regions: striatum (*F* = 15.080, *p* = 0.001, partial *η* ^2^ = 0.485, Figure 5A), thalamus (*F* = 15.217, *p* = 0.001, partial *η* ^2^ = 0.487, Figure 5B), hypothalamus (*F* = 13.376, *p* = 0.002, partial *η* ^2^ = 0.455, Figure 5C), brainstem (*F* = 15.199, *p* = 0.001, partial *η* ^2^ = 0.487, Figure 5D), and midbrain (*F* = 21.675, *p* < 0.001, partial *η* ^2^ = 0.575, Figure 5E), but had no significant effect in male mice. In the control group, male mice exhibited higher SUV_mean_ than female mice: striatum (*F* = 20.922, *p* < 0.001, partial *η* ^2^ = 0.567, Figure 5A), thalamus (*F* = 28.714, *p* < 0.001, partial *η* ^2^ = 0.642, Figure 5B), hypothalamus (*F* = 4.520, *p* = 0.049, partial *η* ^2^ = 0.220, Figure 5C), brainstem (*F* = 10.583, *p* = 0.005, partial *η* ^2^ = 0.398, Figure 5D), and midbrain (*F* = 28.627, *p* < 0.001, partial *η* ^2^ = 0.641, Figure 5E), while no sex differences were observed in the T4 group.

There was no significant T4 × sex interaction in the SUV_mean_ of cortex, hippocampus, and amygdala. About the SUV_mean_ of cortex, highly significant main effect of T4 (*F* = 13.131, *p* = 0.002, partial *η* ^2^ = 0.451) with higher SUV_mean_ in the T4 group (0.375 ± 0.014) than the control group (0.304 ± 0.014; Bonferroni‐adjusted *p* = 0.002, Figure 5F), and highly significant main effect of sex (*F* = 13.984, *p* = 0.002, partial *η* ^2^ = 0.466) with males showing higher SUV_mean_ (0.376 ± 0.014) than females (0.303 ± 0.014; Bonferroni‐adjusted *p* = 0.002). About the SUV_mean_ of hippocampus, highly significant main effect of T4 (*F* = 11.514, *p* = 0.004, partial *η* ^2^ = 0.418) with higher SUV_mean_ in the T4 group (0.276 ± 0.011) than the control group (0.223 ± 0.011; Bonferroni‐adjusted *p* = 0.004, Figure 5G), and highly significant main effect of sex (*F* = 19.365, *p *< 0.001, partial *η* ^2^ = 0.548) with males exhibiting higher SUV_mean_ (0.284 ± 0.011) than females (0.215 ± 0.011; Bonferroni‐adjusted *p *< 0.001). About the SUV_mean_ of amygdala, significant main effect of T4 (*F* = 8.512, *p* = 0.010, partial *η* ^2^ = 0.347) with higher SUV_mean_ in the T4 group (0.580 ± 0.033) than the control group (0.442 ± 0.033; Bonferroni‐adjusted *p* = 0.010, Figure 5H), while the main effect of sex was not significant.

### 3.5 No Significant Correlation Between Glial Reactivity and Behavioral Performance in Hyperthyroid Mice

Significant differences were observed between the control and T4 group in immobility time, number of platform crossings, and target quadrant distance. Accordingly, we further analyzed the correlations of SUV_mean_ as well as GFAP and IBA‐1 densities with these three behavioral indicators (immobility time, number of platform crossings, and target quadrant distance) in T4 mice.

Pearson correlation analysis revealed that no significant correlations were found between cortical GFAP/IBA‐1, hippocampal GFAP/IBA‐1 densities, and all behavioral indicators after FDR correction (Table 2).

**TABLE 2:** Correlation analysis results between average density of GFAP/IBA‐1 in the cortex/hippocampus and behavioral indicators.

Average density of GFAP/IBA‐1	Behavioral indicator	Correlation coefficient (*r*)	Raw *p*‐value	FDR‐corrected *p*‐value
GFAP_ cortex	Immobility time	−0.115	0.751	0.987
GFAP_ cortex	Number of platform crossings	0.264	0.461	0.987
GFAP_ cortex	Target quadrant distance	0.309	0.385	0.9573
IBA‐1_ cortex	Immobility time	−0.442	0.2	0.9573
IBA‐1_ cortex	Number of platform crossings	−0.172	0.635	0.987
IBA‐1_ cortex	Target quadrant distance	0.03	0.934	0.987
GFAP_ hippocampus	Immobility time	−0.855	0.002	0.052
GFAP_ hippocampus	Number of platform crossings	−0.448	0.194	0.9573
GFAP_ hippocampus	Target quadrant distance	−0.624	0.054	0.702
IBA‐1_ hippocampus	Immobility time	0.188	0.603	0.987
IBA‐1_ hippocampus	Number of platform crossings	−0.35	0.322	0.9573
IBA‐1_ hippocampus	Target quadrant distance	−0.333	0.347	0.9573

*Note*: Statistical significance was set at FDR‐corrected *p*‐value < 0.05.

Abbreviations: GFAP, glial fibrillary acidic protein; IBA‐1, ionized calcium‐binding adapter molecule 1.

After FDR correction, SUV_mean_ in all detected brain regions had no statistically significant correlations with the behavioral indicators (Table 3).

**TABLE 3:** Correlation analysis results between SUV in different brain regions and behavioral indicators.

Brain region	Behavioral indicator	Correlation coefficient (*r*)	Raw *p*‐value	FDR‐corrected *p*‐value
Striatum	Immobility time	0.0436	0.9445	0.9942
Striatum	Number of platform crossings	0.0974	0.8185	0.9921
Striatum	Target quadrant distance	0.4443	0.2701	0.7203
Amygdala	Immobility time	0.0496	0.9369	1.0
Amygdala	Number of platform crossings	0.3295	0.4255	0.851
Amygdala	Target quadrant distance	0.754	0.0381	0.508
Hippocampus	Immobility time	0.1894	0.7733	0.9666
Hippocampus	Number of platform crossings	0.5739	0.1369	0.9127
Hippocampus	Target quadrant distance	0.4892	0.2267	0.6477
Cortex	Immobility time	0.3452	0.4012	0.8446
Cortex	Number of platform crossings	0.4218	0.3125	0.7812
Cortex	Target quadrant distance	0.5123	0.2015	0.6717
Thalamus	Immobility time	−0.0433	0.9448	0.969
Thalamus	Number of platform crossings	0.2446	0.5593	0.8286
Thalamus	Target quadrant distance	0.734	0.0381	0.508
Hypothalamus	Immobility time	−0.043	0.945	0.945
Hypothalamus	Number of platform crossings	0.245	0.559	0.86
Hypothalamus	Target quadrant distance	0.734	0.038	0.76
Brain stem	Immobility time	−0.083	0.894	0.9933
Brain stem	Number of platform crossings	0.26	0.534	0.8544
Brain stem	Target quadrant distance	0.774	0.024	0.96
Midbrain	Immobility time	−0.091	0.884	1.0
Midbrain	Number of platform crossings	0.124	0.77	0.9935
Midbrain	Target quadrant distance	0.646	0.083	0.664

*Note*: Statistical significance was set at FDR‐corrected *p*‐value < 0.05.

## 4 Discussion

Our study demonstrates that T4 induces widespread glial reactivity in the mouse brain, supported by increased IBA‐1 and GFAP expression and elevated SUV_mean_ on in vivo imaging. PET/CT revealed distinct sex‐ and region‐specific effects: T4 markedly increased SUV in striatum, thalamus, hypothalamus, brainstem, and midbrain in female mice only, while in cortex, hippocampus, and amygdala, T4 increased SUV in both sexes. Hyperthyroid mice showed increased locomotor and exploratory behavior, especially in males, without cognitive or depressive‐like deficits. Though no correlation was detected between glial reactivity and the noncognitive and non‐depressive‐like behavioral alterations, these findings suggest the importance of sex and regional stratification in investigating TH's central effects.

In this study, we integrated immunofluorescence and PET imaging to provide multidimensional evidence of TH‐induced glial reactivity. Consistent with previous studies (Noda et al. 2016; Rousseau et al. 2020), we observed significant upregulation of IBA‐1 and GFAP in the hippocampus and cortex of hyperthyroid mice, pathologically confirming reactive microglia and astrocytes. This was corroborated by increased SUV in the same regions, validating TSPO PET as an imaging biomarker for TH's central effects on the glial reactivity (Loggia 2024; Boniek and Malamut 2025; Mueller et al. 2023).

While cortical and hippocampal glial reactivity was consistently induced by TH across sexes, we observed sexual dimorphism in two forms. First, PET imaging revealed a significant T4 × sex interaction in subcortical regions (striatum, thalamus, hypothalamus, brainstem, midbrain), where T4 increased SUV exclusively in females. This regional sensitivity may be related to the inherent sexual dimorphism of these nuclei, as subcortical brain regions undergo prominent sexual differentiation and exhibit persistent structural and functional sex differences (Swaab 2007). In addition, Villa et al. (2018) reported that female microglia display distinct phenotypes and stronger responses to hormonal stimuli than males. Second, GFAP expression displayed region‐specific sex dimorphism: higher cortical density in males, higher hippocampal density in females. The elevated hippocampal GFAP density observed in female mice was in line with prior work that documented sex‐related disparities in hippocampal GFAP immunoreactivity among adult rats (Arias et al. 2009). In contrast, our observation of higher cortical GFAP density in males is not fully consistent with a recent translatome analysis of neocortical astroglia (Rurak et al. 2022). That study reported that sex differences in cortical astroglial maturation and GFAP expression peak at early postnatal stages and become milder in adulthood. This discrepancy may reflect differences in species or brain regions examined between studies. Collectively, these findings demonstrate that while TH broadly leads to glial reactivity, the regional and cellular manifestations of this response are profoundly shaped by sex.

Although hyperthyroid mice did not exhibit overt depressive‐like or cognitive deficits in this study, they displayed distinct behavioral alterations, including shortened immobility time and increased platform crossings, specifically in males. These changes are likely attributable to elevated locomotor and exploratory activity induced by TH (Hochbaum et al. 2024; López 2024). As reported by Hochbaum et al. (2024), TH directly remodels cortical circuits and boosts excitatory drive to promote exploratory and locomotor behaviors in males, while coordinating whole‐body metabolic state.

In the present study, correlation analysis revealed no significant association between glial reactivity and the observed noncognitive and non‐depressive‐like behavioral alterations. Two major factors may account for this observation. First, the increased locomotor and exploratory behavior resulted from TH remodeling of cortical circuits, by activating cell‐type‐specific transcriptional programs in the frontal cortex of adult mice. T3‐induced transcriptional changes in astrocytes are associated with synaptic support and metabolic maintenance, suggesting a permissive or supportive role in neuronal circuit remodeling rather than serving as the direct drivers of exploratory behavior (Hochbaum et al. 2024). Second, neither the expression levels of GFAP and IBA‐1 nor the SUV of PET imaging reflect the full spectrum of glial functional status. They represent only one aspect of glial reactivity and fail to fully capture associated changes such as neurotransmitter and metabolic alterations, proinflammatory factor release, and microglial phenotype (Sofroniew 2009; Hass and Barnstable 2016).

Several limitations inherent to the present study should be recognized. First, considered the small sample size, correlational analyses were performed on the merged cohort without stratification by sex, which could have obscured potential sex‐specific relationships between glial reactivity and TH‐related behavioral changes. Validation in larger cohorts is therefore warranted. Second, although our behavioral battery assessed depressive‐like behaviors, spatial learning and memory, and reward preference, it did not include assessments of anxiety‐like behaviors (e.g., elevated plus maze) or social interaction, precluding a comprehensive evaluation of TH's effects on a broader range of brain functions. Third, the use of SUV, a conventional semi‐quantitative index in PET, provides a measure of total tracer uptake but cannot completely rule out the impact of nonspecific factors that are not associated with glial reactivity.

## 5 Conclusions

In conclusion, our study provides integrated PET/CT and pathological evidence of sex‐ and region‐specific glial reactivity in hyperthyroid mice. No significant correlations between glial reactivity and the observed noncognitive and non‐depressive‐like behavioral alterations. These results underscore the importance of sex and regional stratification in investigating TH's central effects, and provide preliminary in vivo imaging evidence supporting the utility of ^1^ ^8^F‐DPA714 PET imaging in detecting hyperthyroidism‐associated glial reactivity.

## Author Contributions

**Qianqian Xue**: conceptualization, investigation, funding acquisition, writing – original draft, writing – review and editing, visualization, methodology, data curation. **Chao Wang**: investigation. **Xiuting Lin**: investigation. **Weibing Miao**: funding acquisition, writing – review and editing, project administration, supervision. **Yuhong Ye**: investigation. **Zefang Lin**: conceptualization, investigation, writing – original draft, writing – review and editing, methodology, visualization, data curation. **Yaofeng Wang**: conceptualization, methodology, data curation. **Yun Yang**: investigation.

## Funding

The article is supported by the Natural Science Foundation of Fujian Province (No. 2023J01590), National Natural Science Foundation of China (No. 82472012), Fujian Provincial Clinical Key Specialty Construction Project (2023SZDZK‐HYXK), and Startup Fund for Scientific Research of Fujian Medical University (No. 2022QH1057).

## Ethics Statement

All experimental procedures and animal care protocols were approved by the Experimental Animal Ethics Committee of Fujian Medical University (IACUC FJMU2023‐Y‐1000).

## Conflicts of Interest

The authors declare no conflicts of interest.

## Data Availability

The datasets used and/or analyzed during the current study are available from the corresponding author on reasonable request.
